# Are All Types of Expertise Created Equal? Car Experts Use Different Spatial Frequency Scales for Subordinate Categorization of Cars and Faces

**DOI:** 10.1371/journal.pone.0067024

**Published:** 2013-06-24

**Authors:** Assaf Harel, Shlomo Bentin

**Affiliations:** 1 Laboratory of Brain and Cognition, National Institute of Mental Health, National Institutes of Health, Bethesda, Maryland, United States of America; 2 Department of Psychology, Hebrew University of Jerusalem, Jerusalem, Israel; 3 Center of Neural Computation, Hebrew University of Jerusalem, Jerusalem, Israel; University of British Columbia, Canada

## Abstract

A much-debated question in object recognition is whether expertise for faces and expertise for non-face objects utilize common perceptual information. We investigated this issue by assessing the diagnostic information required for different types of expertise. Specifically, we asked whether face categorization and expert car categorization at the subordinate level relies on the same spatial frequency (SF) scales. Fifteen car experts and fifteen novices performed a category verification task with spatially filtered images of faces, cars, and airplanes. Images were categorized based on their basic (e.g. “car”) and subordinate level (e.g. “Japanese car”) identity. The effect of expertise was not evident when objects were categorized at the basic level. However, when the car experts categorized faces and cars at the subordinate level, the two types of expertise required different kinds of SF information. Subordinate categorization of faces relied on low SFs more than on high SFs, whereas subordinate expert car categorization relied on high SFs more than on low SFs. These findings suggest that expertise in the recognition of objects and faces do not utilize the same type of information. Rather, different types of expertise require different types of diagnostic visual information.

## Introduction

An enduring question in object recognition is what defines expert perceptual processes and how different they are from regular, everyday object recognition. This question was amply explored in the domain of face processing, a domain of natural expertise in all human adults. Faces form a highly homogenous set of stimuli with a very similar spatial configuration of parts. Therefore, discriminating between individual faces and extracting other relevant information from them should be, in theory, a difficult perceptual task. Nonetheless, humans are extremely adept in recognizing individual faces and categorizing faces along many other subordinate dimensions such as race or gender. This remarkable ability has been attributed by some to specifically tuned neural mechanisms distinguished by behavioral [1], electrophysiological [2], and neuroimaging [3]–[4] markers. Others considered face perception as an extreme manifestation of perceptual expertise [5]–[6], which may be generalized to objects other than faces, specifically to objects that form a visually homogenous category with a prototypical part configuration [7]–[8]. The latter view challenges face specificity suggesting that processing characteristics usually attributed to faces are a general expression of expert visual processing rather than a peculiarity of face recognition. The present study addresses this contended issue by investigating the type of visual information utilized by experts while recognizing objects of expertise, and whether such information is different from expert processing of faces, on the one hand, and from processing objects outside the domain of expertise, on the other hand.

Several lines of evidence suggest similarities between the manifestation of face expertise and accumulated expertise for other real-world objects. First, similar to the individuation of faces, experts tend to recognize objects from their domain of expertise at a subordinate-level and do so with relative ease, in spite of the high visual homogeneity of the exemplars within a category. This tendency has been termed “the subordinate shift” [9] and has been demonstrated by similar category verification speed for both basic and subordinate levels [10]–[12]. The subordinate shift has been reported in experts with various object categories [9], [13]–[15] as well as with faces [16]–[17]. Furthermore, it has been suggested that objects of expertise, similar to faces, are processed in a holistic fashion, that is, by integrating the parts of the object rather than processing them independently from one another [7], [18]. These two properties, the subordinate shift and holistic processing, are considered the hallmark properties of object expertise [19]. Second, several neuroimaging studies have shown that the fusiform face area (FFA), a putative face-selective area, is activated more strongly by objects of expertise than by other objects [8], [20]–[21], albeit other studies failed to find such an effect [22]–[25]. Finally, event related potential (ERP) studies showed that the N170, an early negative occipitotemporal component that indexes face processing (increasing in amplitude in response to faces compared to objects) may be modulated by expertise to non-face objects [26]–[30].

Together, the studies presented above were considered support for the notion that faces and objects of expertise share common perceptual and neural mechanisms [31]. However, this view is intensely debated [19], [32] and several studies suggested that face expertise and object expertise might utilize visual information differently [33]–[35]. These studies describe manipulations that impair face processing but not the processing of objects of expertise. For example, Yue and colleagues [35] have shown that whereas matching of faces was influenced by spatial frequency (SF) manipulations, the matching of laboratory-created objects of expertise (“blobs”) by trained experts was unaffected by identical SF manipulations. This occurred even though the blobs were specifically designed to resemble faces in their surface properties and physical similarity. These findings putatively support a computational model of face and object recognition proposed by Biederman and colleagues in which objects of expertise (as well as regular objects) are represented based on invariant features, free of SF information whereas faces are represented holistically by a set of spatially distributed V1-like filters that are sensitive to orientation and SF content [36]–[37].

While the results of Yue and colleagues [35] are revealing, their psychological reality is limited because they are based on artificial rather than real-world objects (for a similar argument, see [38]). Moreover, artificial objects, unlike real-world objects lack semantic representations. This point is critical, as expertise could be characterized by the ability to access relevant and meaningful semantic information that is not available to non-experts [39], [40]. Therefore, when studying face and object expertise it is necessary to explore not only what kind of perceptual information is utilized by observers that vary in their domain and level of expertise, but also how this information interacts with the expert’s knowledge and recognition goals. In fact, the lack of sensitivity to SF manipulations in blob experts may have been confined to situations that do not require the usage of specific SF scale information, such as basic-level categorization [41].

Supporting this conjecture, Harel and Bentin [42] found that categorization of faces and objects requires different SF scales and, critically, that this differential utilization of SF information depends on the level of categorization. Specifically, categorization of faces at both basic and subordinate level was impaired by filtering out low SFs from the image but was not affected when high SFs were filtered out. In contrast, filtering out low or high SFs equally impaired categorization of airplanes at the basic-level, but subordinate categorization of airplanes was more impaired by filtering out high SFs. This pattern implies that the use of perceptual information in the image is determined by multiple factors, primarily stimulus category, expertise with that category, and also the level of the categorization. However, in that study the processing of faces was not contrasted with the processing of other objects of expertise and, therefore we could not establish whether the differences in using SFs are specific to face processing or a general manifestation of expertise.

To investigate whether expertise for faces and other objects entails the usage of the same type of spatial frequency information, in the present study we compared the categorization of faces, cars, and airplanes by car experts and novices while explicitly controlling for level of categorization. This design allowed us to manipulate the factors that we suggest might affect the utilization of SF information while processing visual images: the level as well as type of expertise, and the level of categorization. The primary theoretical interest concerned the utilization of SF information within the car experts group at the level at which their expertise might be expected to manifest, that is, at the subordinate level. Expanding our previous findings, if face and car expertise utilize the same type of perceptual information, we would predict that car categorization in car experts would show a similar sensitivity to SF manipulations as that found for face expertise. In this case, categorization of cars by car experts at the subordinate level should be more hampered by the filtering of low rather than high SFs. Alternatively, if expert car categorization is based on different diagnostic information than face expertise, a different pattern of SF utilization should emerge. One possibility, for example, is that expert car categorization will rely more on high rather than low SFs, resembling everyday object recognition. Thus, the current design enabled us to test whether expert recognition involves the usage of common diagnostic information across categories, or whether each type of expertise is manifested differently.

## Materials and Methods

### Participants

Fifteen car experts (all males, 21–42 years, M = 26.4) and fifteen undergraduate students from the Hebrew University of Jerusalem (all males, 19–31 years, M = 25.2) participated in the study, which was conducted at the Hebrew University of Jerusalem, Israel. They signed an informed consent according to the requirements of the Ethics Committee of the Hebrew University and were paid for participation. Approval was obtained from the Hebrew University ethics committee.

The car experts were recruited among volunteers who responded to a message posted in car forums on the Internet. To assess their expertise, the candidates performed a same-different car model recognition task inspired by Gauthier et al. [8] (see details below), and an additional semantic task that was aimed at assessing the extent of their knowledge and familiarity with the cars presented in the model recognition task (not reported here). Expertise in the current study was determined by scoring an accuracy level of 83% or above in the car recognition task. Furthermore, all self-defined experts displayed extensive knowledge about car models in the semantic task. Importantly, all “experts” reported a life-long interest in cars and displayed extensive knowledge about the cars presented in this study.

All the participants completed the expertise testing procedure (same-different recognition tests with cars and airplanes) in a separate session prior to the main experimental session.

### Expertise Testing Procedures

On each trial of the same-different car-model recognition test, participants determined whether two cars presented sequentially (for 500 ms each with 500 ms ISI) were of the same model (e.g. “Honda Civic” or not). The two cars in each trial were always of the same make (e.g. Honda), but were physically different, as they differed in year of production, color, angle and direction of presentation. Thus, while for ‘different model’ trials the two images were of the same car-maker but different models (e.g. VW Golf and VW Passat), for ‘same model’ trials the two images depicted two cars of the same model but differed physically The experiment consisted of 80 trials (40 ‘same model’ trials, 40 ‘different model’ trials), which were based on 160 different car images. No identical pairs or images were repeated throughout the experiment. The car images were of frequently encountered models from recent years. To assure that the car expertise displayed by the car experts was category-specific, all participants performed an analog experiment in which they were instructed to match images of passenger airplanes. The passenger planes experiment was prepared and displayed in the same manner as the car experiment (e.g. based on 160 different airplane images). The order of the trials within each experiment varied across participants (for further details on the expert selection procedure, see [24]).

Formal comparison of the experts’ performance with that of novices was based on mixed-model, two-way ANOVA with expertise (experts/non-experts) as a between-subjects factor and object-category (airplane/car) as a within-subjects factor. Accuracy rate (d’) was the dependent variable. This analysis showed a significant interaction between the two factors (F(1,21) = 50.76, p<.001). As expected, experts were highly more accurate when recognizing cars (Mean d′ = 2.49, range = 2.04 to 3.58) compared to airplanes (Mean d′ = 0.70, range = 0.12 to 1.16) while the performance of novices was similar for cars and airplanes (Mean d′ = 0.45, range = −0.24 to 1.04 and Mean d′ = 0.41, range = −0.14 to 0.71, respectively).

### Experimental Stimuli

The stimuli in the main experiment were 80 images of female faces in front view, half Chinese and half Israeli, 80 images of cars in side view, half of European makers and half of Japanese makers and 80 images of airplanes in side view, half combat jets and half passenger airliners. The models of cars that we selected are frequently encountered in Israel.

**Figure 1:**
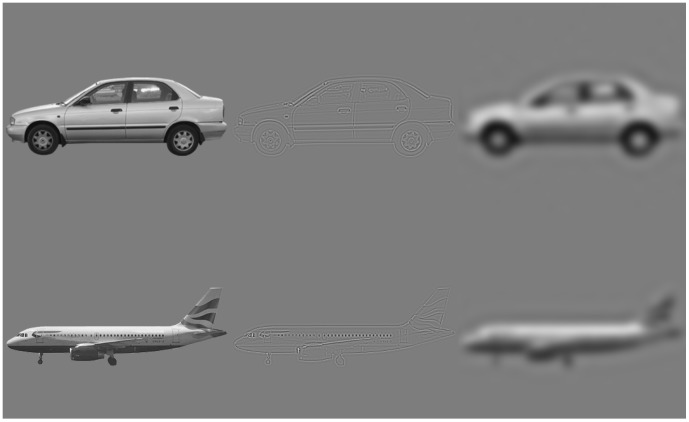
Examples of stimuli used in the experiment. For cars (top row), Japanese makers are presented, and for airplanes (bottom row), civil airliners are represented. Faces are not presented for privacy reasons. Stimuli are presented in the different spatial frequency scale conditions: BB (left column), LP (center column) and HP (right column). Note that for presentation purposes the stimuli in the HP condition are presented using a slightly lowered threshold.

**Figure 2:**
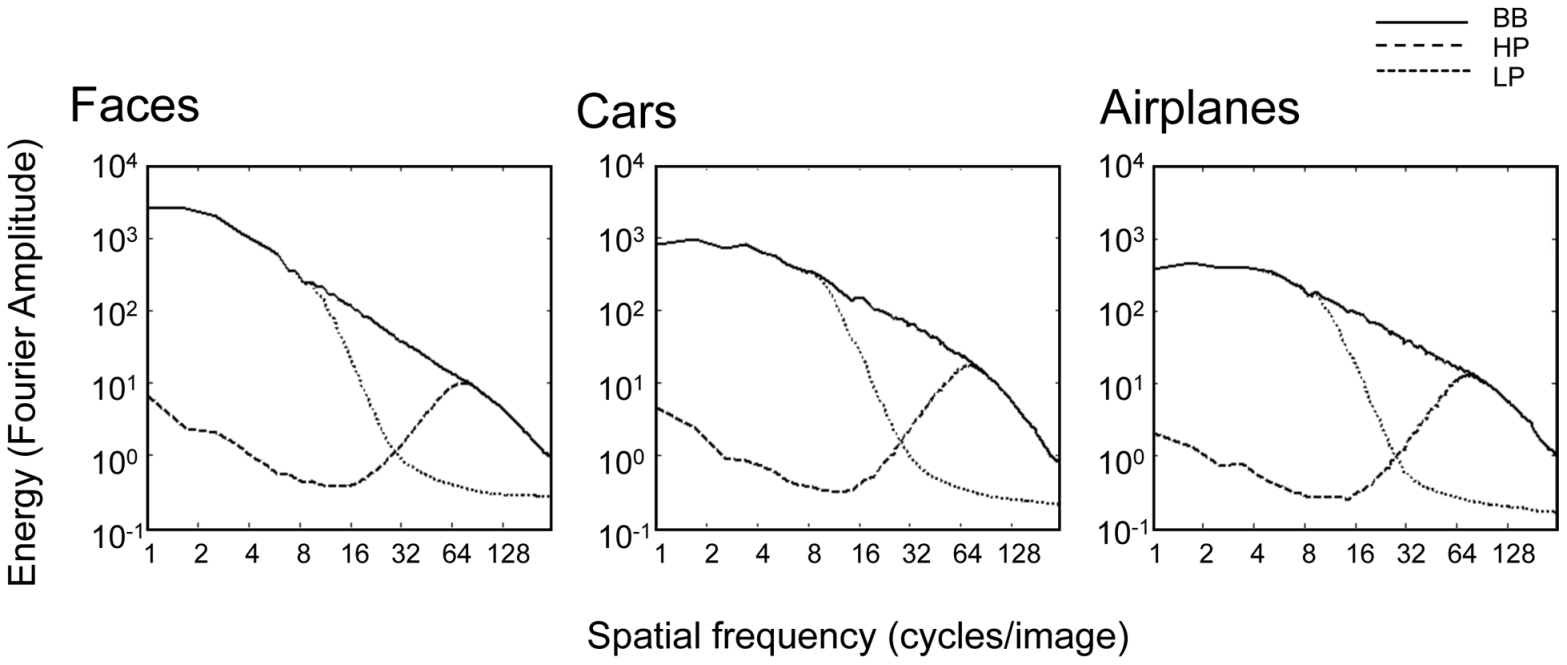
Mean magnitude versus frequency plot of the spatial frequency spectra of the spatially filtered stimuli in the three object categories (see text for details).

All images where 360×360 pixels, which seen from a distance of 70 cm subtended a square of 9.9°×9.9° at the center of the visual field. The object image size, mean luminance and RMS contrast were equated across categories. The background of all the objects was a uniform gray equated to the mean objects luminance. The original, broadband (BB) images were spatially filtered using a Butterworth filter with an exponent of 4. The low-pass (LP) and high-pass (HP) filter cutoff corners were 1 cycle/degree (∼10 cycles/image) and 6.5 cycles/degree (65 cycles/image), respectively. All together there were 720 different images, 240 in each spatial filter condition. Examples of the stimuli in the different spatial filters can be seen in Figure 1. The values of the HP and LP filters were set to match previous studies (e.g. [43]). In order to rule out effects induced by possible differences between categories in the energy of different frequency bands after filtering, we measured the energy of the stimuli across for each category in each spatial frequency scale (Figure 2). Each image in the stimuli set was Fourier transformed and its DC component was set to zero. A rotational average of the Fourier amplitudes of each radius in the plane was calculated. This yielded for each image a distribution of energy across orientations as a function of spatial frequency. The resulting spectra were then averaged separately for each experimental condition (irrespective of the task-related categorization level): faces BB, faces LP, faces HP, cars BB, cars LP, cars HP, airplanes BB, airplanes LP, airplanes HP. As can be seen in Figure 2, the values were roughly equivalent across categories for each type of spatial filter.

### Experiment Design and Procedure

Participants performed a category verification task in two consecutive sessions. A trial started with the presentation of an object category label presented for 500 ms at the center of the screen. The label was followed by a fixation cross for 250 ms and then by an image of an object exposed for 300 ms, and a blank screen for 250 ms, after which a question mark appeared. The question mark was the signal for the participant to initiate a response (The delayed response procedure was imposed by the concomitant recording of ERPs. These data will be reported elsewhere). Randomly selected inter-trial intervals of 500, 800, 1000 ms separated the next trial from the response. Participants were instructed to indicate by pressing one of two buttons whether the object matched the category label or not. Accuracy rates and reaction times (RTs) were recorded. RTs were measured from the onset of the question mark. As the imposed delay of the responses precluded drawing strong conclusions, the RT data is not further reported. Nonetheless, it is worth mentioning that the pattern of results replicated the RTs in our previous study in which they were measured from stimulus onset [42].

Throughout the experiment, the level of categorization was blocked. Thus, basic level (face, car, or airplane) and subordinate level (Chinese or Israeli face, European or Japanese car, civil airliner or combat jet) category labels were presented in two separate sessions administered consecutively, using the same stimuli. Note that although each stimulus image appeared twice, they were presented in two different sessions and separated by a large number of trials, hence precluding any potential priming effects. The order of the blocks was counterbalanced across participants. A session consisted of five blocks, each consisting of 144 trials. The nine stimulus types (three categories and three spatial filter conditions) were *mixed* in each block and presented in random order. In half of the trials the image corresponded with the preceding category label and in the other half the image did not correspond with the preceding label. In the subordinate level, images in the mismatch trials were from the same basic-level category as the category label. For example, the category label “Chinese face” was followed by an image of an Israeli face. In the basic level condition, mismatch images were from the two object categories other than the object category label. For example, following the label “car”, an image of an airplane or an image of a face appeared with equal probability. Each block of the experiment was preceded by instructions and a training session of 72 trials comprising of all the experimental conditions in equal proportion.

## Results

To assess the accuracy of categorization performance, a measure of sensitivity (d’) was calculated for each experimental condition. The d’s were calculated by collating the responses of the participants for each trial (i.e., match/mismatch between the category label and the image) and comparing it with the required correct responses (match/mismatch between the category label and the image). A mixed-model ANOVA with Group (car experts/novices) as a between-subjects factor and Category (faces/cars/airplanes), Categorization Level (basic/subordinate) and SF scale (BB/HP/LP) as within-subjects factors was performed. The analysis showed a main effect of Group (F(1,28) = 59.30, *MSE* = 160, *p*<.0001), Category: F(2,56) = 67.46, *MSE*<1, p<.0001; Categorization Level: F(1,28) = 160.00, *MSE = *1, *p*<.0001, and SF scale: F(2,56) = 49.26, *MSE<*1, *p*<.0001). All the interactions in the experiment were significant (p<.01) other than the SF×Group interaction (F(2,56) = 1.80, *MSE<*1, *p*>.15).

As noted above, the crucial question outlining the current study is whether car experts utilize SF information in a way that resembles more face categorization or object (airplane) categorization. As expertise in the recognition of both faces and objects is primarily expressed at the subordinate level of categorization, our following analyses focus on subordinate level categorization. Indeed, basic-level categorization performance was highly accurate across all SF conditions, with no significant interaction between SF scale and Category in both the expert (F(4,56)<1.00) and novice group (F(4,56)<1.00) (Table 1). This pattern is consistent with many prior studies using the category verification paradigm (e.g., [17], [41]).

**Table 1:** Basic-level categorization performance of novices and car experts (d’).

	Airplanes			Cars			Faces		
	BB	HP	LP	BB	HP	LP	BB	HP	LP
Novices	3.55	3.48	3.52	3.56	3.66	3.62	3.49	3.76	3.70
	(0.18)	(0.18)	(0.17)	(0.17)	(0.17)	(0.17)	(0.17)	(0.18)	(0.20)
Experts	4.02	3.99	3.84	4.15	4.07	4.10	3.96	3.88	3.91
	(0.14)	(0.17)	(0.19)	(0.14)	(0.17)	(0.17)	(0.18)	(0.12)	(0.16)

Basic-level categorization performance (mean d’) of airplanes, cars and faces at the different spatial frequency scale conditions for the novices and car experts. BB = Broadband images; HP = High-pass filtered images, LP = Low-pass filtered images. Error bars represent SEM.

**Figure 3:**
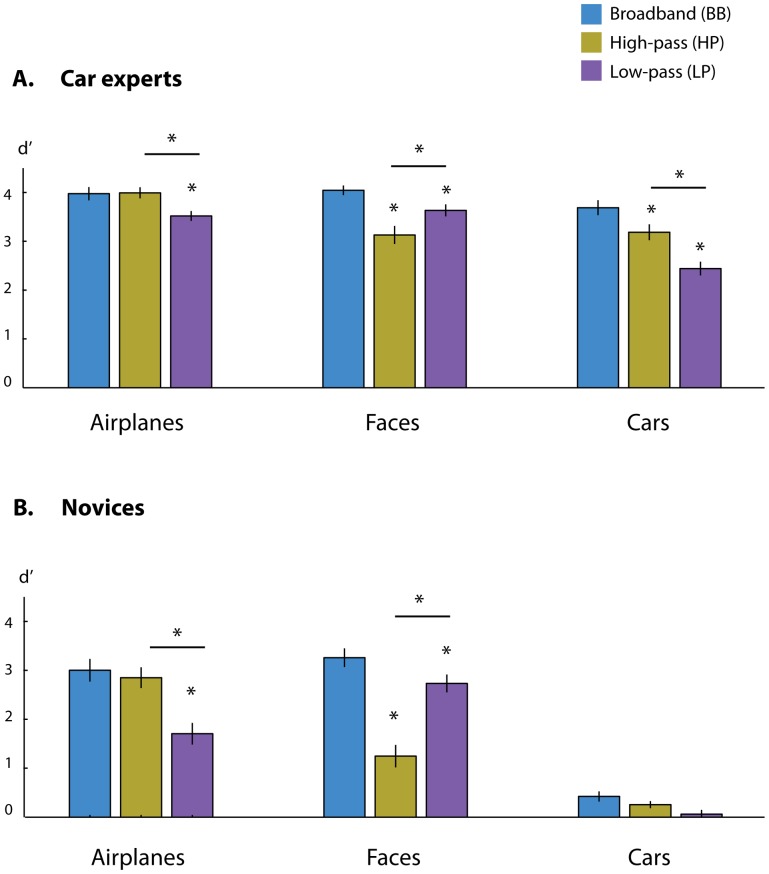
Subordinate categorization performance (mean d’) of the car experts (A) and novices (B). (A) Expert subordinate level categorization of faces, airplanes, and cars at the different SF scale conditions. (B) Novice subordinate level categorization of faces, airplanes, and cars at the different SF scale conditions. BB = Broadband images; HP = High-pass filtered images, LP = Low-pass filtered images. Error bars represent SEM. * denotes significance value of p<0.05.

Stating with the novice group, the current results replicated our previous findings with novices [42] demonstrating that subordinate categorization of faces and airplanes rely on different types of SF scales (novice subordinate car categorization was not further analyzed due to its overall poor performance level (mean d’ = .24, see Figure 3B)). Airplanes and faces showed opposite patterns of SF utilization (F(4,56) = 37.03, *MSE<*1, *p*<.0001; Figure 3B). Subordinate face categorization was significantly lower in its accuracy relative to subordinate airplane categorization (t(14) = 5.26, *p*<.001) when the lower SFs were removed from the image (the HP condition). In contrast, subordinate face categorization was significantly higher than the airplane subordinate categorization (t(14) = 5.33, *p*<.001) when the higher SFs were filtered out from the image (the LP condition).

Having established a double dissociation between faces and objects (airplanes) in utilization of SF information, the following question was whether subordinate car categorization in experts would exhibit a similar sensitivity to SF manipulations as that displayed in subordinate face categorization. As can be seen in Figure 3A, this was not the case. Compared to the BB condition, subordinate categorization of cars by the car experts was more interfered by the removal of *high* SFs (mean difference = 1.25, *p*<.001; Bonferroni corrected) than by the removal of *low* SFs (mean difference = .50, *p*<.05; Bonferroni corrected) whereas subordinate categorization of faces was hampered by the removal of *low* SFs to a greater extent (mean difference relative to the BB condition = .91, *p*<.001; Bonferroni corrected) than by the removal of *high* SFs (mean difference relative to the BB condition = .41, *p*<.05; Bonferroni corrected). A direct comparison of faces and car categorization reveals that when the higher SFs were filtered out from the image (the LP condition) subordinate car categorization was significantly lower than the face subordinate categorization (t(14) = 7.93, *p*<.001). Thus, information usage in expert subordinate car categorization shows a different pattern of sensitivity to SF manipulations relative to that of face subordinate categorization.

The pattern of information usage in the subordinate expert categorization is clearly distinct from that used in face categorization, and resembles in its higher reliance on the higher SFs the subordinate categorization of airplanes. However, this may raise the question of how specific is the pattern of information usage in car expert categorization to the participants’ expertise, or whether it reflects a general category difference in information usage, similar to the difference found between face- and airplane- subordinate categorization. To address this possibility, we compared the subordinate categorization of cars and airplanes under the different SF scales within the expert group. If the effect of car expertise reflects information usage that is general to objects, one should not expect a difference between car and airplane subordinate categorization across the different SFs scales. Alternatively, a difference between airplane and car categorization in experts would imply unique expert processing. To adjudicate between these possibilities, a mixed-model ANOVA with Category (cars/airplanes) and SF scale (BB/HP/LP) as independent variables was performed. Critically, the Category by SF scale was highly significant (F(2,28) = 9.48, *MSE = 1.23, p*<.001), implying a differential usage of SF information for the categorization of cars and airplanes in experts. Follow-up analyses of this interaction showed that car subordinate categorization was more impaired the airplane subordinate categorization when the lower SFs were removed (t(14) = 3.86, *p*<.005), but it was even more impaired when the higher SFs were removed (t(14) = 11.83, *p*<.0001). Thus, subordinate car categorization is unique from airplane categorization, but this difference is quantitative, and not qualitative, with greatest decrease noted when the higher SFs were filtered out. Note that when all frequency scales are contained within the image (BB condition), there was no difference between car and airplane subordinate categorization (t(14) = 1.62, *p*>.13) implying same level of task difficulty for these two baseline conditions (see next).

Finally, the pattern of results for the subordinate categorization in the experts cannot be simply explained by differences between categories resulting from task difficulty, as planned comparisons showed no significant difference in expert baseline performance (BB condition) between the three categories (F(2,28) = 2.45, *MSE<1, p*<.11).

## Discussion

The goal of the present study was to explore how naturally developed expertise for faces and intentionally learned expertise for cars manifest during visual object recognition. To achieve this goal we compared the impact of expertise on the utilization of image-based information (SF scales) for category verification of faces, cars and airplanes while controlling for categorization level. Specifically, our question was whether car expertise and face expertise will utilize the same type of SF information when categorized at a subordinate level.

The present findings revealed that expert subordinate categorization of cars varied as a function of the SF content of the image differently than faces. Whereas for faces the distinction between Chinese and Israelis was less accurate when *low* spatial frequencies were removed from the faces, for cars the distinction between Japanese and European cars by car experts was substantially less accurate when *high* spatial frequencies were removed from the cars. Note that for airplanes, an object category of non-expertise, the subordinate distinction was also worse when *high* spatial frequencies were removed. This pattern of results, (i.e., the difference between car expert recognition and face recognition in SF utilization on the one hand and the putative similarity between car expert recognition and subordinate object recognition, on the other hand) suggest that expertise in the recognition of objects, including faces, does not entail a unifying computational principle. Rather, different types of expertise require different types of diagnostic visual information.

Our choice of subordinate tasks in the current study might seem at first non-intuitive. Expertise in face recognition is usually considered to manifest at the individual exemplar level, that is, discrimination of facial identity. Accordingly, it may be argued that the choice of a race categorization task might not have tapped the “true” nature of face expertise. However, while individuation undoubtedly requires expertise, it may not be the only type of face expertise. As proposed by Johnson and Mervis in their seminal study of bird and fish experts [40], expertise can be expressed at multiple levels of abstraction along the basic level to individual exemplar level continuum. For example, bird experts can easily distinguish between loons, ducks, and grebes (subordinate level) but at the same time distinguish between a teal and mallard (sub-subordinate), as well as between a redhead duck and a canvasback duck (sub-sub-subordinate). In other words, while the individual exemplar level may serve as the entry point (particularly in faces, see [17]; but see [16]), there may not necessarily be a single “subordinate” level that captures expertise in its entirety. The rationale for the use of the race categorization task in the current study was our elaborate effort to match as much as possible the face and the object expertise tasks. The subordinate distinction between European and Japanese cars is non-trivial, and it was designed specifically so that the experts will have to employ their full recognition abilities (albeit at the unexpected consequence of very low performance for the novice group). In terms of categorization hierarchies, the face analog of the car subordinate task is the distinction between Israeli and Chinese faces, as it is more specific than the distinction between objects and faces but less specific than individuation. Similarly, the distinction between a civil airliner and combat jet is more specific than a basic-level distinction, but at the same time does not require identification of individual exemplars. On a more general note, it should be noted that the question of whether the level of specificity along the categorization hierarchy interacts with object category and stimulus information (i.e. what subordinate distinctions would be considered a-priori as “equivalent” when comparing the categorization of different object categories) has not received much attention in the literature, and would greatly benefit from future research [for further discussion, see 41].

In contrast to the dissociation between subordinate categorization of faces and objects of expertise, basic-level categorization was similarly high for both objects of expertise (faces and cars) and for objects outside the domain of expertise (airplanes), in both experts and novices, suggesting that expert-specific utilization of SF information is manifest primarily at the subordinate level. While this pattern might reflect ceiling effects, previous research suggests that this caveat is not necessarily warranted. Since basic level categorization is based on the recognition of the general shape of the object [12], [44] it is usually considered to be insensitive to spatial filtering manipulations, as the general shape of the object is retained in both low-pass and high-pass filters [41], [45]–[46]. Thus, the current pattern of results for the basic level condition is congruent with this notion as well as with other studies in which RTs were analyzed in addition to performance [41]–[42]. Further, this pattern was also evident in a study in which noise was added to spatially filtered images to circumvent potential ceiling effects [41]. Finally, previous training studies of expertise showed that superior expert performance was manifest when the participants were required to make subordinate level discriminations, but not when they were required to make basic level ones [15], [47].

The current findings are not in accord with previous studies of expertise that proposed that expertise with different types of objects involves the usage of similar perceptual information (cf. [31]). This view was based on studies showing that face processing is influenced by the concomitant processing of objects of expertise. Thus, Gauthier and colleagues showed that the holistic processing of faces in the context of cars was more interfered with when the cars were presented in a normal orientation than when holistic processing of the cars was hampered presenting them with their top half upside-down. Importantly, the magnitude of interference correlated with the level of car expertise. This was considered evidence that, for car experts but not for novices, cars tap the same holistic processing mechanism as faces [48]. Similarly, the face-sensitive N170 potential, decreased in amplitude when car experts viewed faces concurrently-presented with cars (relative to concurrently presenting two faces). This effect was not observed with novices [27]. These results were proposed as evidence for a functional dependence between face and object expertise, particularly in the usage of holistic information (see also [7]). However, other studies of expertise in object recognition failed to find holistic effects using known experimental paradigms from the face literature, such as inversion effect, the composite effect and contrast reversal ([33]–[34] but see [49]). These studies challenged the notion that expertise with objects and face expertise utilize the same type of perceptual (holistic) information. At the same time, however, these studies did not address the actual process of information utilization that occurs in expertise, leaving the question of what is the nature of the diagnostic information in expertise unanswered.

The present study fills in this gap by demonstrating that under more or less equal recognition goals, car expertise relies primarily on high SFs whereas face expertise relies on low SFs. Apparently, when details are not required, expert object recognition involves only the processing of the overall shape; this process was used for both faces and cars. However, when finer discriminations are required object recognition is dominated by the need to extract diagnostic features, which are different for different categories [50]. The diagnostic information required to distinguish between Chinese and Israeli (Caucasian) faces was contained in low SFs whereas the distinction between Japanese and European cars was based on information contained mainly in high SFs. Although a simple relationship between low and high SFs and holistic/featural processing of visual information is contentious [51]–[54] details are evidently absent from low-pass filtered images whereas the holistic and texture information, although might be present, are more difficult to discern when low SFs are removed. Therefore, the present findings suggest that subordinate face categorization (at least by race) requires information about the configuration of parts and texture, whereas expert car categorization requires detailed information about diagnostic parts. Hence, notwithstanding controversies about how holistic and part information is contained in different SF scales, when fine distinctions are required, experts differ in the type of information utilized during visual processing. Finally, it is important to note that our use of “high” or “low” SFs in the current work is in a purely relative sense, and is used only to infer the *differential* usage of SF scales. Put differently, the primary concern of the present study is not what are the absolute SF scales diagnostic for recognition of different object categories (as measured in cycles per object), but rather whether the categorization of faces and cars (in experts) is *equally* impaired by filtering out particular SF scales. In this respect, our choice of specific cut-off values was strictly utilitarian: We used SF cut-off values that have been shown to be relevant for face and car recognition in a prior study [43].

It is also important to note that the utilization of details was not specific to subordinate categorization of objects of expertise. The distinction between the two subcategories of airplanes has also been based on details conveyed by high SFs. Since the participants were not airplane experts the selective use of high SFs for this distinction suggests that even non-experts could extract diagnostic information selectively, based on their knowledge. This pattern raises the possibility that the manifestation of expertise for cars in the present study also reflected knowing what the diagnostic features of the two subordinate categories were. For the subordinate categorization of airplanes, this information is probably common knowledge (see the long line of windows characteristic to civil airliners) whereas for cars the knowledge of the diagnostic features is probably acquired through experience. A recent study investigating the long-term structural cortical changes that are associated with increasing experience in car recognition provides support for this conjecture [39]. We found that experience in car recognition was positively correlated with increasing gray matter density in prefrontal cortex. Based on this finding, we hypothesized that acquiring perceptual expertise in a specific category is accompanied by the acquisition of vast personal visual knowledge related to that category, which leads to the formation of enriched and distinctive visual representations that are accessed and processed by prefrontal regions (see also [24]). Finally, it should be emphasized that while there are similarities in the usage of information for the categorization of objects of expertise (cars) and objects of non-expertise (airplanes), this does not necessarily mean that the two processes are identical. Comparing the categorization of cars and airplanes by the experts showed that a differential modulation of the two categories by SF scale. While categorization of both categories was most influenced by the removal of the higher SFs, the cars showed a greater decrement than the airplanes, indicating a greater reliance on details for categorization. Notably, this distinction between objects of expertise and regular objects implies that the difference we found between faces and cars in experts cannot be reduced to a general category effect (i.e., reflecting a similar SF profile difference as the difference between faces and airplanes), further suggesting that it is the combination of experience and category, which drives the difference between the processing of faces and non-face objects of expertise.

### Conclusions

The present study demonstrated that when expertise is needed to discriminate between members of a homogenous category, the diagnostic information extracted from the image is determined by the specific stimulus category. For faces, the diagnostic information, at least for race distinctions was at the lower end of the SF spectrum. For cars, the diagnostic information resided in details; hence, the removal of high SFs impaired performance. This pattern lead us to conclude that the impact of expertise on visual processing is based on acquired knowledge about category specific diagnostic details that are required for discriminating between similar exemplars of subordinate categories. Whereas for faces this knowledge might be applied by default [42] for other objects of expertise it is applied only when it is instrumental, such as in difficult subordinate categorization tasks.
